# Evaluation of the potential defensive strategy against Influenza A in cell line models

**DOI:** 10.12688/f1000research.13496.2

**Published:** 2018-05-16

**Authors:** Ekaterina Antonova, Olga Glazova, Anna Gaponova, Aykaz Eremyan, Natalya Grebenkina, Svetlana Zvereva, Natalya Volkova, Pavel Volchkov

**Affiliations:** 1 Moscow Institute of Physics and Technology, Dolgoprudny, Moscow Region, 141701, Russian Federation; 2 Ernst Institute of Animal Husbandry, Podolsk Municipal District, Moscow Region, 142132, Russian Federation

**Keywords:** sialidases, Influenza A, defence strategy, exogenous expression, α(2, 3)-sialylation

## Abstract

**Background:** Influenza virus can cause both seasonal infections and unpredictable pandemics. Rapidly evolving avian H5N1 and  H7N9 viruses have a potential pandemic threat for humans. Since avian Influenza can be transmitted by domestic birds, serving as a key link between wild birds and humans, an effective measure to control the influenza transmission would be eradication of the infection in poultry. It is known that the virus penetrates into the cell through binding with the terminal oligosaccharides - sialic acids (SA) - on the cell surfaces. Removal of SA might be a potential antiviral strategy. An approach to developing chicken lines that are resistant to influenza viruses could be the creation of genetically modified birds. Thus it is necessary to select a gene that provides defense to influenza. Here we have expressed in cells a range of exogenous sialidases and estimated their activity and specificity towards SA residues.

**Methods:** Several bacterial, viral and human sialidases were tested. We adopted bacterial sialidases from *Salmonella *and *Actinomyces *for expression on the cell surface by fusing catalytic domains with transmembrane domains. We also selected Influenza A/PuertoRico/8/34/H1N1 neuraminidase and human membrane sialidase ( *hNeu3*) genes. Lectin binding assay was used for estimation of a α (2,3)-sialylation level by fluorescent microscopy and FACS.

**Results:** We compared sialidases from bacteria, Influenza virus and human. Sialidases from *Salmonella *and Influenza A neuraminidase effectively cleaved α (2-3)-SA receptors. Viral neuraminidase demonstrated a higher activity. Sialidases from *Actinomyces* and *hNeu3* did not show any activity against α (2-3) SA under physiological conditions.

**Conclusion**: Our results demonstrated that sialidases with different specificity and activity can be selected as genes providing antiviral defence. Combining chosen sialidases with different activity together with tissue-specific promoters would provide an optimal level of desialylation. Tissue specific expression of the sialidases could protect domestic birds from infection.

## Introduction

The global influenza pandemic remains a real threat. Avian influenza viruses H5N1 and H7N9 have killed millions of birds, including domestic poultry, causing enormous financial losses. 860 cases of disease in human population has been detected since 2003 and 454 of them with fatal outcome (see http://www.who.int/influenza/human_animal_interface/2018_03_02_tableH5N1.pdf?ua=1). The risk of a new pandemic of viral infection remains high. Seasonal flu vaccination is used as a traditional way to protect individuals in an outbreak nearby location, however it has a lot of limitations. Also it is difficult to reduce the infection in bird population that are natural transmitters of the Influenza. Therefore, it is critical to develop alternative approaches to prevent influenza infection. An alternative way to reduce the infection could be the creation of genetically modified domestic birds. It will decrease the risk of the infection spread, because domestic birds are known to transmit the infection acting as an intermediary between wild ducks to humans ( Kim *et al*., 2009). It is necessary to select gene-candidates for developing the approach.

The best way to prevent the infection is to impede the virus from entering the cell. Influenza virus hemagglutinin (HA) provides attachment to the host cell that leads to fusion between the virion envelope and the host cell membrane ( Skehel & Wiley, 2000). Surface sialic acid (SA) residues are host cell epitopes that are recognized by influenza virus A and B ( Ito, 2000). SA are a family of nine-carbon acidic monosaccharides that naturally terminate sugar chains attached to the proteins on the cell surface ( Varki *et al*., 2009). Neuraminidase (NA) - the second major surface antigen - is an exoglycosidase, or saildase, that cleaves SA from cell membrane glycolipids and glycoproteins, thus destroying the recognition epitopes on the surface of the host cell for the viral receptor HA. NA activity helps viral particles to penetrate through mucous secretions that are rich in sialic acids, to reach the target cells in the airway epithelium ( Palese *et al*., 1974). The role of the enzyme in facilitating release of newly formed viral particles from the infected cell surface and preventing aggregation of the viral particles was experimentally confirmed ( Matrosovich *et al*., 2004; Palese *et al*., 1974). There are known antiviral agents - oseltamivir and zanamivir - that inhibit NA, blocking the release of virus particles from infected cells ( Moscona, 2005; Varghese, 1999). Sialidases have also demonstrated to be effective inhibitors of Influenza virus infection *in vitro*. It has been shown that cells treated with *Vibrio cholerae* or *Micromonospora viridifaciens* bacterial sialidase are resistant to influenza virus infection ( Air & Laver, 1995; Bergelson *et al*., 1982; Griffin *et al*., 1983; Stray *et al*., 2000).

**Figure 1.**
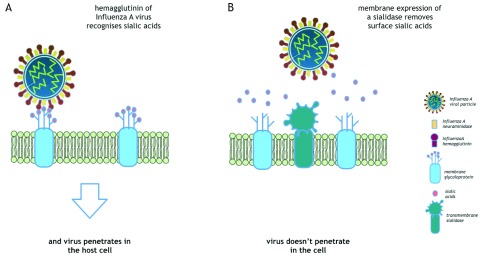
Schematic of the antiviral strategy. Influenza A virus penetrates into the host cell through the binding with the host cell surface sialic acids. ( **B**) Removal of sialic acids through exogenous sialidase expression protects cells from viral infection.

We focused on exogenous expression of sialidases as a defense strategy against influenza infection ( Figure 1). Here we show the protective effect of exogenous expression of different sialidases. The range of exogenous sialidases has diverse activity and specificity towards SA residues. Tissue-specific expression of sialidases in transgenic poultry might protect domestic birds against Influenza virus.

## Methods

### Cloning of sialidases

***Viral neuraminidase.*** Viral neuraminidase from human Influenza A virus was amplified from the plasmid pNAHA (Plasmid # 44169; Addgene, Cambridge, MA, USA) that encodes the neuraminidase gene of Influenza A / Puerto Rico / 8/34, subtype N1. The amplified product was inserted by restriction cloning in the lentiviral transfer plasmid based on a pHAGE backbone (#24526, Addgene) after the CMV promoter. The RFP reporter was cloned in the same reading frame and the resulted construction was pHAGE-CMV-infNA-P2A-RFP ( Supplementary Figure 1A). Additionally the construction RFP-P2A-infNA was cloned in the same vector having RFP and infNA in reverse order ( Supplementary Figure 1A).

### Bacterial sialidases

***Sialidases from Salmonella typhimurium and Actinomyces viscosus.*** Synthesis of the codon-optimized bacterial sialidase from *Salmonella typhimurium* (Uniprot / P29768, 2–382aa) and the codon-optimized bacterial sialidase from *Actinomyces viscosus* (uniprot / Q59164, 347–631aa) fused with the transmembrane domain of viral neuraminidase (1 – 75 aa) were done in BioCat. The transmembrane domain was added to provide membrane localisation of the enzymes, since sialidase *S.typhimurium* and *A. viscosus* are cytoplasmic. The codon-optimization was done with the IDT tool with the choice of the Gallus gallus genome. The genes were ordered in the standard vector pUC57 and were flanked by the restriction sites NheI and SphI for *S. typhimurium* and XhoI and SphI sites for *A. viscosus*. The genes were excised from the vector pUC57 using the sites and inserted into the pHAGE backbone after the the CMV promoter. As a result, the plasmids pHAGE-CMV-Sal.t.Sia-P2A-RFP and pHAGE-CMV-Act.v.Sia-P2A-RFP have been cloned ( Supplementary Figure 1A).

### Human neuraminidase

The genes coding sialidase 3 (membrane sialidase) in G.gallus and H.sapiens are homologous based on the analysis in HomoloGene (NCBI). We selected human membrane sialidase (Gene ID: 10825) for cloning because there is more information about it compared with other orthologs. The coding exons were amplified from human genomic DNA and overlapped. The resulted product was inserted by restriction cloning in the lentiviral transfer plasmid based on the pHAGE backbone (#24526, Addgene) after CMV promoter. The RFP reporter was cloned in the same reading frame and the resulted construction was pHAGE-CMV-hNeu3-P2A-RFP ( Supplementary Figure 1A).

### Catalytically inactive sialidase

Enzymatically inactive neuraminidase (d_infNA) was taken as a negative control. Two amino acids at positions 262–263 (EE) of the Influenza A virus strain A/Puerto Rico/8/1934 H1N1 are responsible for the substrate binding. The inactive variant has a mutation - E262D ( Huang *et al*., 2008). The mutation was made by two overlapping primers: 5’-GCACCTAATTCTCACTATGAtGAATGTTCCTGTTAC-3’, 5’-CATTCaTCATAGTGAGAATTAGGTGC-3’ that change the codon from GAG to GAT (The changed nucleotides for the overlapping primers are marked as lowercase letters). Primers were made by Evrogen (Moscow, Russia). The changed PCR product was then cloned into the same backbone. The resulting construction was pHAGE-CMV-d_infNA-P2A-RFP

### Tet-on system, design and cloning

Tet-on system was set up to make transcription of the gene of interest to be inducible ( Gossen & Bujard, 1992; Gossen *et al*., 1995). rtTA (tetracycline transactivator), CMV promoter, TRE (Tet Response Element) promoter P2A and RFP were amplified from different plasmids. Influenza A neuraminidase and sialidase from *S. typhimurium* were inserted under control of TRE-promoter. Neuraminidase was amplified from the plasmid pNAHA (Plasmid # 44169, Addgene) and sialidase *S. typhimurium* was amplified from pHAGE-CMV-Sal.t.Sia-RFP. All amplified products were cloned in the pHAGE backbone in the following order: TRE-infNA/Sal.t.Sia-P2A-RFP-CMV-rtta by restriction cloning ( Supplementary Figure 1B). The plasmid pTagBFP encoding BFP (blue fluorescent protein) was used for cotransfection to approximately estimate the effectiveness of transfection before addition of the doxycycline.

### Doxycycline induction

Cells were treated with Doxycycline (D9891, Sigma; St Louis, MI, USA) at 24 hours after transfection at a concentration of 0.5µg/ml. Expression of the RFP reporter and activity of genes-enzymes were analyzed at 48 hours after Doxycycline addition.

### Cell cultivation, transfection, microscopy and FACS analysis

MDCK and HEK293 cell lines were cultured according to ATCC recommendations and maintained at 37°C with 5% CO _2_. The cells were transfected with TurboFect reagent (R0531, Thermo Fisher; Waltham, MA, USA) according to the manufacturer recommendations. The fluorescence microscope Axio observer (Zeiss, Oberkochen, Germany) with the standard set of filters was used for visualization of fluorescence. S3e BioRad sorter (Hercules, CA, USA) was used for fluorescence activated cell sorting and flow cytometry. Standard flow cytometry analysis was carried out using FlowJo v10.4 software.

### Statistical analysis

FITC fluorescence in Lectin binding assay was measured by FACS and quantified as an average mean fluorescence intensity (MFI). Values show the means ± SD of triplicate results from representative experiments. Three independent experiments were carried out for each experimental case. Student’s *t*-test was used to determine the level of statistical significance. Statistical analysis was perform in Microsoft Excel 2016.

### Lentiviral production, determination of viral titre and viral transduction of MDCK cell line

Lentivirus packaging was performed according to the described protocol [ see Protocol 1: Lentivirus Packaging by 293T Transfection from CReM].

In order to determine a viral titre, different aliquots of supernatant were added to HEK293 cells in the presence of 4 μg/ml polybrene (Hexadimethrine bromide, H9268, Sigma). Infection of six 10-fold serial dilutions was performed in 96-well plates. The medium was replaced 12 hours after infection. Infection with the same aliquot was used in three repeats. At 48 hours after infection, transduction effectiveness was calculated by FACS analysis. FACS analysis was performed using non-infected cells as a negative control. The cells with 10%–40% RFP-positive were selected for titer calculation. The titer was calculated using the equation: Titer (Transduction Unit/ml) = (cell number in each well on the day of infection × percentage of GFP/RFP positive cells (should be 10%~40%))/(the virus volume used for infection in each well × dilution fold).

The supernatant of the known titre was used for viral transduction of the MDCK cell line. On the first day about 1.0 × 10 ^4^ MDCK cells were seeded into 6-well plates. The cells were incubated 18–20 hours at 37°C in a humidified incubator in an atmosphere of 5–7% CO2. Upon transduction the confluency around 30–50% was estimated. Next, the appropriate volumes of unconcentrated virus and polybrene with concentration 5ug/ml were added to each well. After 48 hours, cells were analyzed using Flow cytometry to define the percentage of infected/fluorescent cells. Lentiviral transduction efficiency was calculated as (the number of RFP-positive cells/the total number of cells counted × 100) and the number of transduced cells (transduction efficiency (%) × the total number of RFP recovered/100).

### Genomic DNA extraction and PCR analysis

Genomic DNA was isolated using Wizard® Genomic DNA Purification Kit (A1120, Promega, Madison, WI, USA) according the manufacturer protocol.

### Lectin binding assay

Lectin binding assay was used for the cell surface SA detection. For lectin binding assay we used *Maackia amurensis* (MA) lectins that are specific for α (2–3)-bound SA. The lectin were conjugated with the FITC fluorophore (21761036-1 (510183), bio-WORLD; Dublin, OH, USA). The assay was made for HEK293 and MDCK cells. The experiment was setup in 48-well plates. The cells expressing a desired construct were washed three times with PBS. Then cells were incubated with MA lectins (1:100) about 1 hour and three times washed with PBS. Hoechst (RRID:AB_2651133, Thermo Fisher) was added for visualization of the nuclei for 5 min at recommended concentration with the subsequent washing with PBS.

## Results

### Transient expression of genetic constructs in HEK293 cell lines

Sialidases vary in enzyme kinetic and substrate specificity to the type of the SA linkage. Several sialidases from bacteria, Influenza virus and vertebrate were selected for our study. We were mostly interested in α(2–3) specialized sialidases, because avian Influenza prefers this type of the linkage ( Ito, 2000). However the broad substrate specificity was also an area of interest. Regarding enzyme kinetics, sialidases with various levels of activity were useful for us in order to combine them with tissue-specific promoters and find an optimal level of desialylation of the cell surface. This optimal level prevents penetration of the virus into the cell and does not affect cell function.

Substrate specificity of bacterial sialidases is very diverse, in terms of type of SA linkages and enzyme kinetics. We selected two different bacterial sialidases: *Salmonella typhimurium* sialidase, which specializes in cleavage of α(2–3) SA residues ( Hoyer *et al.*, 1991; Rogerieux *et al.*, 1993) and *Actinomyces viscosus* sialidase, which has a substrate specificity to both α (2–3) and α (2–6), but preferentially cleaves the α (2–6) linkage ( Teufel *et al.*, 1989). We used sequence of the catalytic domains of the sialidases and added to their N-terminal sequences the transmembrane domain with the stem loop of Influenza NA transmembrane protein in order to target the proteins to the membrane and demonstrate its catalytic activity outside of the cell.

The human Influenza neuraminidase (sialidase) has been taken for analysis as a broad-spectrum sialidase. Unlike human HA that preferentially recognises α (2–6), the viral NA of H1N1 has enzymatic activity to both α(2–6) and α(2–3) types of linkages, however the rate of cleavage of α(2–6) linked SA is lower than α(2–3) ( Solano *et al*., 2017).

Exogenous overexpression of a vertebrate membrane sialidase also would be interesting to analyze. The sialidases are poorly investigated, but some experimental data exists for human membrane neuraminidase hNeu3 ( Monti *et al.*, 2000; Zhang *et al.*, 2010) and the enzyme was selected for cloning.

**Figure 2.**
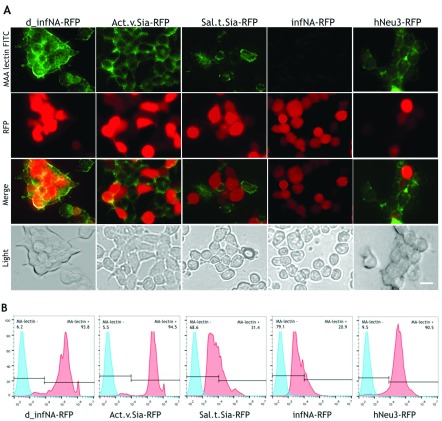
Evaluation of sialidase activity in the HEK293 cell line. Genetic constructs were transiently expressed in the HEK293 cell line, and sialidase activity was evaluated using a lectin binding assay with FITC-labelled *Maackia amurensis* lectins. The plasmid with catalytically inactive neuraminidase was used as the negative control. ( **A**) Fluorescent microscopy analysis. The boxes on the panel are zoomed views of an image fragment. The scale bar = 10 um. ( **B**) FACS analysis. Histograms show the fluorescence intensity of the cells incubated with FITC-labelled lectins. Blue - fluorescence intensity of the cells without incubation with lectins (negative control), pink - fluorescence intensity of the cells incubated with lectins.

All cloned genetic constructs were firstly tested by transient expression in the HEK293 cell line that has glycosylation patterns including α (2–3) and α (2–6) SA linkages ( Picanco-Castro *et al.*, 2013). All plasmids had the RFP reporter to mark the cells that express a sialidase. The construction coding catalytically inactive neuraminidase d_infNA-RFP was taken as the negative control. Using lectin binding assays it has been shown that the cells expressing the sialidase from *S. typhimurium* have decreased level of SA. Expression of the sialidase from *A. viscosus* did not affect the α (2–3) sialylation level, probably because it is more specific to the α (2,6) SA linkages. However, it has previously been shown that this sialidase effectively removes both α (2,3)- and α (2,6)-linked SA from the cell surface ( Malakhov *et al.*, 2006). Human neuraminidase hNEU3 also has not shown activity towards α (2–3)-linked SA. Probably, it might be related to unsuitable pH conditions. Cultivation in DMEM in 5% CO _2_ provides pH in range of 7,6–7,7. Earlier, hNeu3 activity was demonstrated in the pH range of 2.8–6.6 ( Monti *et al.*, 2000). However we do not demonstrate here the membrane localisation of the protein, thus we can not be sure in its proper localisation on the cell surface. In the case of influenza A neuraminidase, sialylation level decreased not only in RFP-positive cells, but also in the RFP-negative cells, which are not supposed to express the enzyme ( Figure 2A). According to the flow cytometry analysis, the most significant α (2–3)-linked SA cleavage was in the case of Influenza A neuraminidase expression ( Figure 2B). On the contrary, it is known that the sialidase from *S.typhimurium* is more active than viral neuraminidase N1. It could be explained by slightly different protein folding of sialidase from Salmonella when transmembrane domain and stem loop are added. In the case of viral neuraminidase we used unchanged sequence of natural protein.

Representative histograms from three independent repeats are shown. Lectin binding assay was quantified as fluorescence and calculated as a mean fluorescence intensity - MFI (±SD): d_infNA-RFP - 103.437 (±3.568), Act.v.Sia-RFP - 181.401(±7.988), Sal.t.Sia-RFP - 7.341(±1.323), infNA-RFP - 4.725(±0.983), hNeu3 - 27.379(±2.231).

### Inducible expression of genetic constructs in HEK293 cell lines

A responsive transcription of a sialidase gene gives an opportunity to induce the gene expression in response to a molecule addition. In our case we used Tetracycline-Controlled Transcriptional Activation where transcription is reversibly turned on in the presence of the antibiotic tetracycline derivative doxycycline. The method can be a useful model for studying viral infection in a cell culture and especially for use in genetically modified organisms, because constitutive exogenous gene expression could have a potential harm.

**Figure 3.**
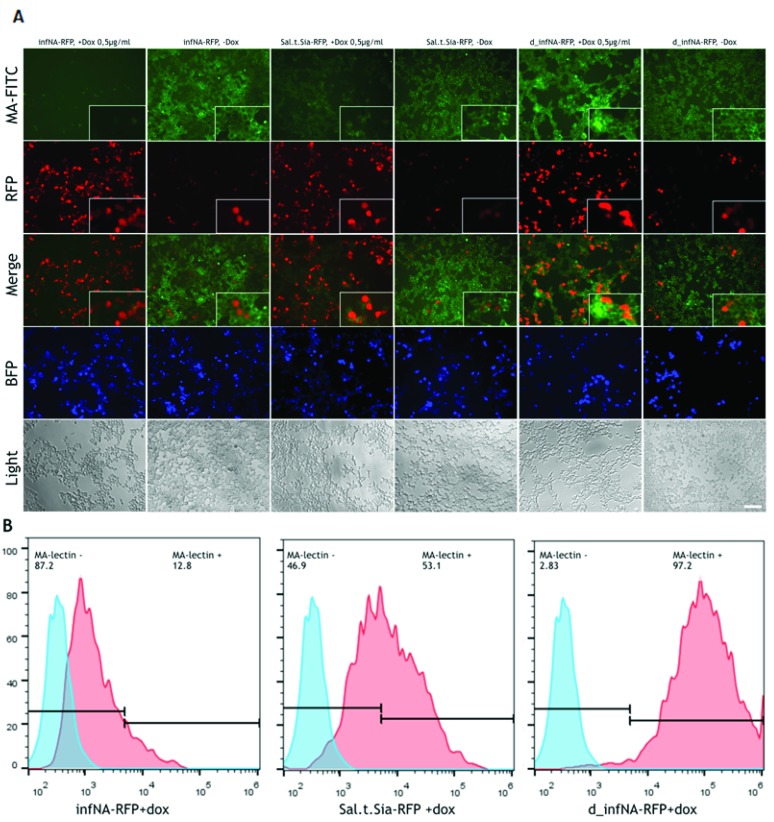
Doxycycline-inducible expression of *S. typhimurium* sialidase and Influenza A neuraminidase in the HEK293 cell line. Gene expression was evaluated in the HEK293 cell line after doxycycline induction, using a lectin binding assay with FITC-labelled *Maackia amurensis* lectins. The plasmid with catalytically inactive neuraminidase was used as the negative control. ( **A**) The plasmid coding BFP was used to estimate the effectiveness of transfection. Fluorescent microscopy analysis. The boxes on the panel are zoomed views of an image fragment. Scale bar = 50 um. ( **B**) FACS analysis. Histograms show the fluorescence intensity of the cells incubated with FITC-labelled lectins. Blue - fluorescence intensity of the cells without incubation with lectins (negative control), pink - fluorescence intensity of the cells incubated with lectins.

We selected only sialidases that demonstrated significant α (2–3) catalytic activity in the previous experiment with transient expression under CMV promoter (infNA-RFP and Sal.t.Sia-RFP) for tetracycline-inducible expression. The plasmid coding BFP under constitutive CMV promoter was used for estimation of transfection effectiveness. It was shown that upon treatment of cells with doxycycline the sialidases expression switched on and as a result the α (2–3) sialylation level decreased ( Figure 3A, B). From flow cytometry analysis representative histograms from three repeats are shown. MFI (±SD) was calculated: infNA-RFP, +dox - 1.468 (±0.439), Sal.t.Sia-RFP, +dox - 6.437(±1.043), d_infNA-RFP, +dox - 83.849(±2.983).

### Lentiviral transduction of MDCK cell lines

The MDCK cell line is used for propagation of influenza viruses. We transduced MDCK cells with lentiviruses. The expressing constructs were integrated into the genome by lentiviral transduction to obtain cell lines that stably express exogenous genes coding for different sialidases. The integration was confirmed with PCR ( Supplementary Figure 2 and Supplementary Figure 3) using primers from Table 1. The effect of expression of sialidases was studied by testing the reactivity of cells with SA linkage-specific lectins.

**Figure 4.**
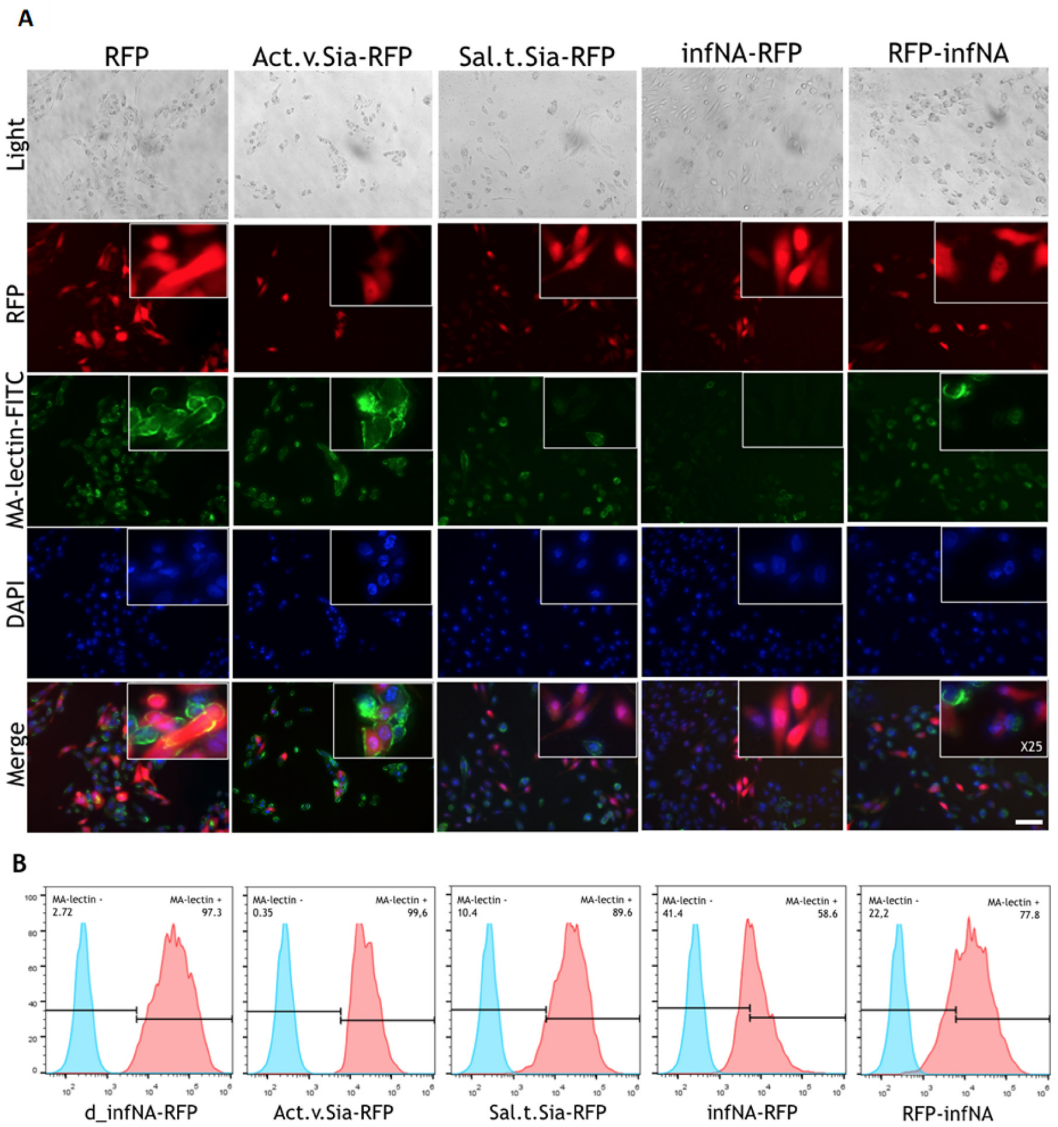
Evaluation of sialidase activity in the MDCK cell line. MDCK cells were transduced with lentiviruses. Lectin binding assay was made with FITC-labelled *Maackia amurensis* lectins. The plasmid with catalytically inactive neuraminidase was used as the negative control. ( **A**) Fluorescent microscopy analysis. The boxes on the panel are zoomed views of an image fragment. Scale bar = 50 um. ( **B**) FACS analysis. Histograms show the fluorescence intensity of the cells incubated with FITC-labelled lectins. Blue - fluorescence intensity of the cells without incubation with lectins (negative control), pink - fluorescence intensity of the cells incubated with lectins.

The results confirmed the previously obtained data with the HEK293 cell line. The cells expressing *S.typhimurium* sialidase and Influenza A neuraminidase had decreased level of the surface α (2–3) SA. However, the decreased level in the MDCK was less significant compared with the HEK293 cell line ( Figure 4B). Representative histograms from three technical repeats are shown. MFI was calculated: d_infNA-RFP - 38.120(±2.322), Act.v.Sia-RFP - 22.750(±3.175), Sal.t.Sia-RFP - 21.732(±4.598), infNA-RFP - 7.290(±1.934), RFP-NA - 12.798(±3.453). Similarly with the experiments on the HEK293 cell line, α (2–3) SA were absent not only in the RFP-positive cells but in the RFP-negative cells as well in the case of infNA-RFP expression. A possible explanation of the evidence could be a ‘slipstream’ translocation in a polycistronic vector of the P2A downstream protein without a signal of localisation that is RFP in our case ( de Felipe *et al.*, 2010). An alternatively explanation being when a ribosome encounters 2A within an open reading frame the synthesis of a specific peptide bond could be “skipped”. This results in termination of translation at the end of 2A peptide ( Donnelly *et al.*, 2001). It means that RFP is not expressed and can explain the absence of SA in some RFP-negative cells. In the case of improper translation of RFP the lentivirus titre determined by FACS could be underestimated. In result, the cells were infected by larger quantity of viral particles and desialylation was more prominent. The effects were not observed in the case of other sialidases. Usage of a longer sequence for P2A (with a favourable upstream sequence composition) or modifying the order of proteins could solve the problem. When we changed the order of the RFP and the infNA sequences (RFP-P2A-infNA), the result of lectin binding assay was as expected: RFP-positive cells showed decreased level of α (2–3) SA ( Figure 4A). Nevertheless, in the case of inverted position of the neuraminidase the enzyme activity was less pronounced. It can be explained by the fact that a protein located at the second position can be slightly less expressed in a polycistronic construct with a 2A peptide ( Liu *et al.*, 2017). In the case of the *S. typhimurium* sialidase expression (Sal.t.Sia-RFP) the 'slipstream’ effect was not observed.

**Table 1.** Primers used to confirm the presence of exogenous insertion.

Name of insertion	Forward primer	Reverse primer
Sal.t.Sia-RFP	5’-AGCGCCACCATGAACCCGAACC-3’	5’- CAATTAAGTTTGTGCCCCAGTTTGC-3’
Act.v.Sia-RFP	5’GGATCCGCTAGCCGCCACCATGAAC-3’	5’- CAATTAAGTTTGTGCCCCAGTTTGC-3’
infNA-RFP	5’-TGGGCTATATACAGCAAAGAC-3’	5’- CAATTAAGTTTGTGCCCCAGTTTGC-3’
RFP-NA	5’-CATGGTGTCTAAGGGCGAAGAGC-3’	5’- CGCCTACTTGTCAATGCTGAATGGCAAC-3’
RFP	5’-CATGGTGTCTAAGGGCGAAGAGC-3’	5’-CAATTAAGTTTGTGCCCCAGTTTGC-3’

## Discussion

Sialic acid is the receptor of Influenza A virus ( Matrosovich *et al.*, 2013). Therefore the removal of SA from the cell surface might be a powerful defence strategy against Influenza virus. Previously, a recombinant sialidase (DAS181) has already shown its antiviral effectivity in the cell culture ( Malakhov *et al.*, 2006) and *in vivo*, using a mouse model (intranasal injection of the recombinant fusion protein - DAS181) ( Belser *et al.*, 2007). Thus, the principal possibility of the usage of membrane-anchored sialidase as antiviral defence has been demonstrated. In the current study we reviewed a variety of sialidases from different sources and compared their activity. We showed that expression of *Salmonella typhimurium* sialidase and human Influenza A neuraminidase on the cellular membrane effectively removed α (2–3)-sialic residues on the cell surface under physiological conditions. The viral neuraminidase had higher cleavage activity against α (2–3)-linked SA than sialidase from *S. typhimurium*.

We are planning to use a sialidase gene as a defensive strategy to create genetically modified domestic birds resistant to Influenza infection. However there are several concerns to use the strategy. The first is that influenza virus receptors may be different from SA, thus, sialidase expression may be ineffective. It has been reported that the virus binding could still occur in the MDCK cell line desialylated with addition in the medium *Micromonospora viridifaciens* sialidase ( Stray *et al.*, 2000). The authors suggested that influenza virus infection can result from SA–independent receptors, either directly or in a multistage process. The presence of SA may enhance virus binding to the cell surface to increase interaction with secondary receptors to mediate entry. High multiplicity of infection has an increased requirement for SA thus desialylation will inhibit the virus amplification. Although the HA of a human H1N1 strain was shown to bind other glycoconjugates ( Rapoport *et al.*, 2006), the exact receptors to enter into the cell have not been found. Stray et al. used NA-deficient virus in the research which was obtained in a laboratory. It is unlikely that such virus would exist in nature. Probably, the barrier to efficient infection and transmission between organisms is due to inefficient use or expression of a yet unidentified entry mediator.

Another concern of the stable expression of sialidases is disruption of physiological functions required for proper glycosylation ( Gutierrez *et al.*, 1987; Michalek *et al.*, 1988; Pangburn *et al.*, 2000; Varki, 1992). The probable consequences of constitutive expression of membrane sialidase *in vivo* are not known. It has been shown that overexpression of the human ortholog *NEU3* membrane sialidase under the β-actin promoter in transgenic mice resulted insulin-resistant diabetes mellitus ( Sasaki *et al*., 2003). The authors focused on insulin signaling only. However at least the data shows that the constitutive membrane sialidase expression was not lethal for mice.

We are going to reduce a probable negative impact on organism using tissue-specific expression of the selected gene. It is necessary to predict a potential impact on the epithelium of intestine and trachea in vivo. There are no similar genetic experiments. However the consequences of tissue-specific expression could be considered from the researches where local desialylation was done. It has been shown that intranasal sialidase treatment does not affect the properties of respiratory mucus, nor did it affect the normal mucus transport activity on ciliated epithelium ( King *et al*., 1974; Meyer *et al*., 1975). Some authors showed that Influenza infection is associated with an increased risk of secondary infection by *Streptococcus pneumoniae* ( van der Sluijs *et al.*, 2006). There are concerns that, probably, tissue-specific expression of sialidases would result in a greater chance of bacterial infection in the respiratory tract due to exposure of its binding sites that were masked by terminal SA. At the same time it has been demonstrated that the binding sites for the bacteria already exist on the normal respiratory surface and sialidase treatment does not introduce *de novo* bacterial adhesion sites. Also the risk of infection arises due to damage of mucus and epithelial cells. But it is not clear whether the secondary *S. pneumoniae* infection happens due to viral NA activity or from a secondary effects of influenza virus intracellular amplification. Influenza infection in the respiratory tract causes much deeper changes to the epithelium than treatment of the surface of the epithelial cells with a sialidase, because Influenza infection in humans and in mice results in vast exposure of the ciliated epithelial cells down to the basal cells and the basement membrane ( Plotkowski *et al*.,1986; Walsh *et al*., 1961). Thus, the most likely the increased *S. pneumoniae* infection is the result of airway epithelial damage caused by the virus infection but not just desialylation. In the gastrointestinal tract SA is an abundant sugar residue in mucin that is a key target of intestinal bacteria. Expression of sailidases will release free SA from mucins that could drives intestinal inflammation and infection. For example, elevated levels of free SA in the gut, during and post antibiotic treatments, promoted the expansion of *Clostridium difficile*, that utilises the free monosaccharide. Also sialidase activity can promote the outgrowth of *Escherichia coli* and result in imbalanced microbiota, inflammation, colitis development due to α (2–3)-sialic residues promote expansion of *E. coli* ( Huang *et al*., 2015). Nevertheless, we do not know the probable negative impact on the physiology of avian respiratory and digestive tract. Thus, an optimal level of desialylation can be established in the tissues when combining genetic constructs with varying sialidase activity and tissue-specific promoters in order to avoid disruption of physiological functions in our approach.

Additionally, a gene expression with time limit could prevent a probable negative impact on a living organism. Michael P. Malakhov *et al.* showed that temporary desialylation of respiratory tract during 7 days using recombinant sialidase protein did not cause any symptoms of toxicity or inflammation in ferrets and mice that were not infected by the virus ( Malakhov *et al*., 2006). This *in vivo* data support our idea that temporary inducible expression of a sialidase will not have serious negative physiological effects for transgenic animals. We are planning to use the Tet-on system as a potential model for creation of genetically modified organism that allows to induce a sialidase expression temporary. Transgenic transcription factor rtTA will be expressed under control of a tissue-specific promoter and a sialidase will be expressed under control of TRE promoter. This enables tissue-specific inducible control of the transgene expression and will provide a powerful and flexible resource for studies of influence of sialylation on chicken physiology and on viral infection. The level of sialylation will return to the previous level during a period of time after withdrawal of an inducing agent. The required time depends on the SA turnover rate. The inducing agent can be treated prophylactically to genetically modified birds in an outbreak that can protect livestock and locally stop infection spread. It is known that the inducible system has some limitations. Firstly, it has basal transgene ‘leak’. However the ‘leak’ is weak and in our case it will be restricted by the specific tissues. The potential consequences of the ‘leak’ will be studied. Secondly, the feeding of domestic birds with the doxycycline antibiotic can compromise its safety as biological products. Nonetheless it is a temporary measure that could help to save the stock. The efficacy of our strategy needs to be evaluated to resolve all the concerns.

In conclusion, our current preliminary *in vitro* data indicates that sialidases from *Salmonella typhimurium* and neuraminidase from Influenza A virus could be the potential candidates that provide antiviral defence against avian Influenza virus. Since sialidases target cellular receptors but not a viral gene product, the chance of influenza viruses developing resistance is low.

## Data availability

Data underlying this study is available from Dataverse - doi: 10.7910/DVN/UCDPX3 ( Antonova, 2018).

## Supplementary material

**Supplementary Figure 1A & 1B. Genetic constructs.**

**Supplementary Figure 2. Confirmation of inserted genes in MDCK genome after lentiviral transduction.**

**Supplementary Figure 3. Schematic of integrated constructs with primers for PCR analysis.**
