# Supplementary figures and images for: Evaluation of the potential defensive strategy against Influenza A in cell line models
Source: F1000Res. 2018 May 16;7:206. Originally published 2018 Feb 19. [Version 2] doi: 10.12688/f1000research.13496.2 (PMC6008855; doi:10.12688/f1000research.13496.2)

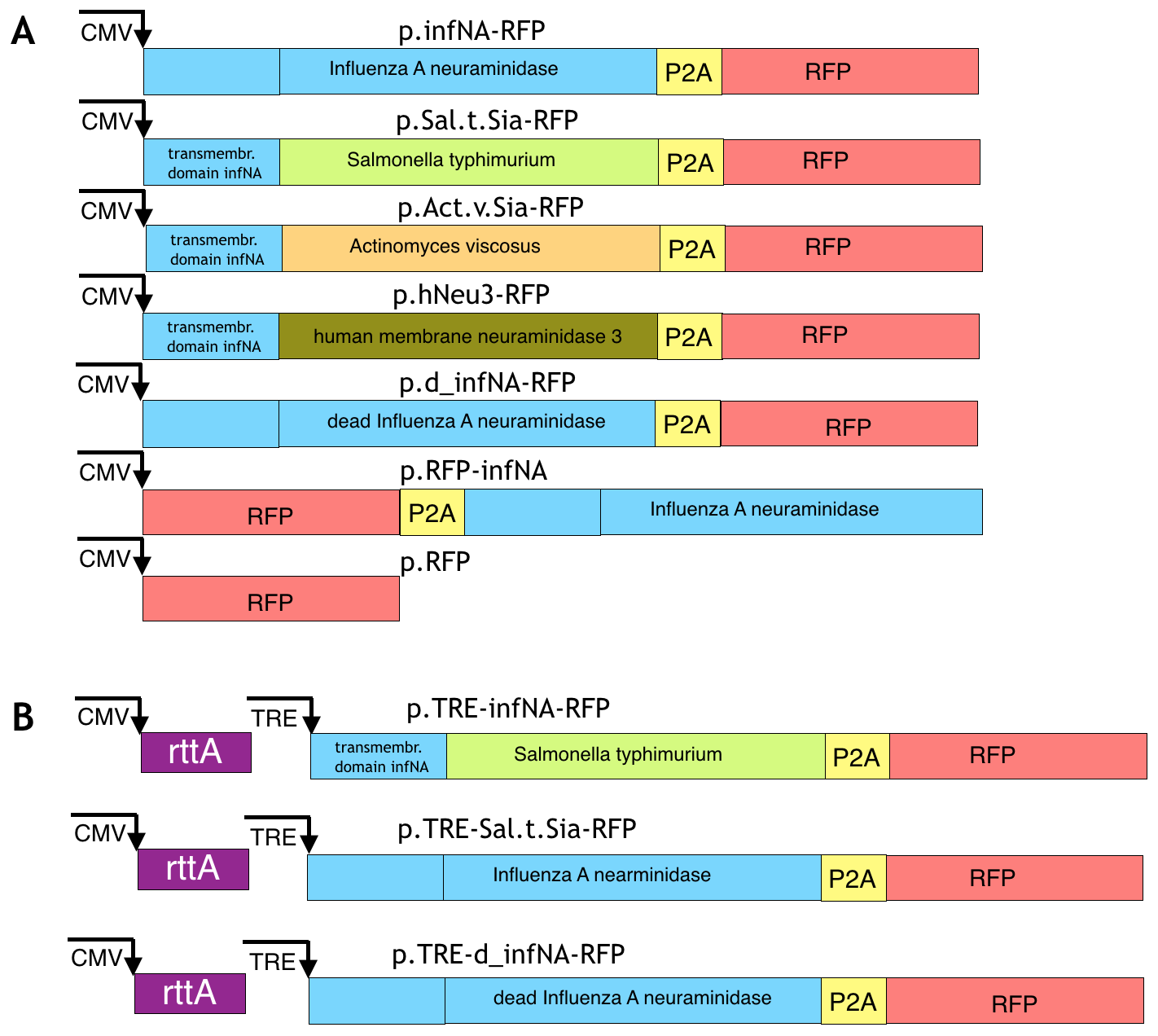

Supplement: Supplementary file 1 [file f1000research-7-16185-s0000.tgz › 3065a4b2-df15-49fb-941d-8ef439964a40.png]

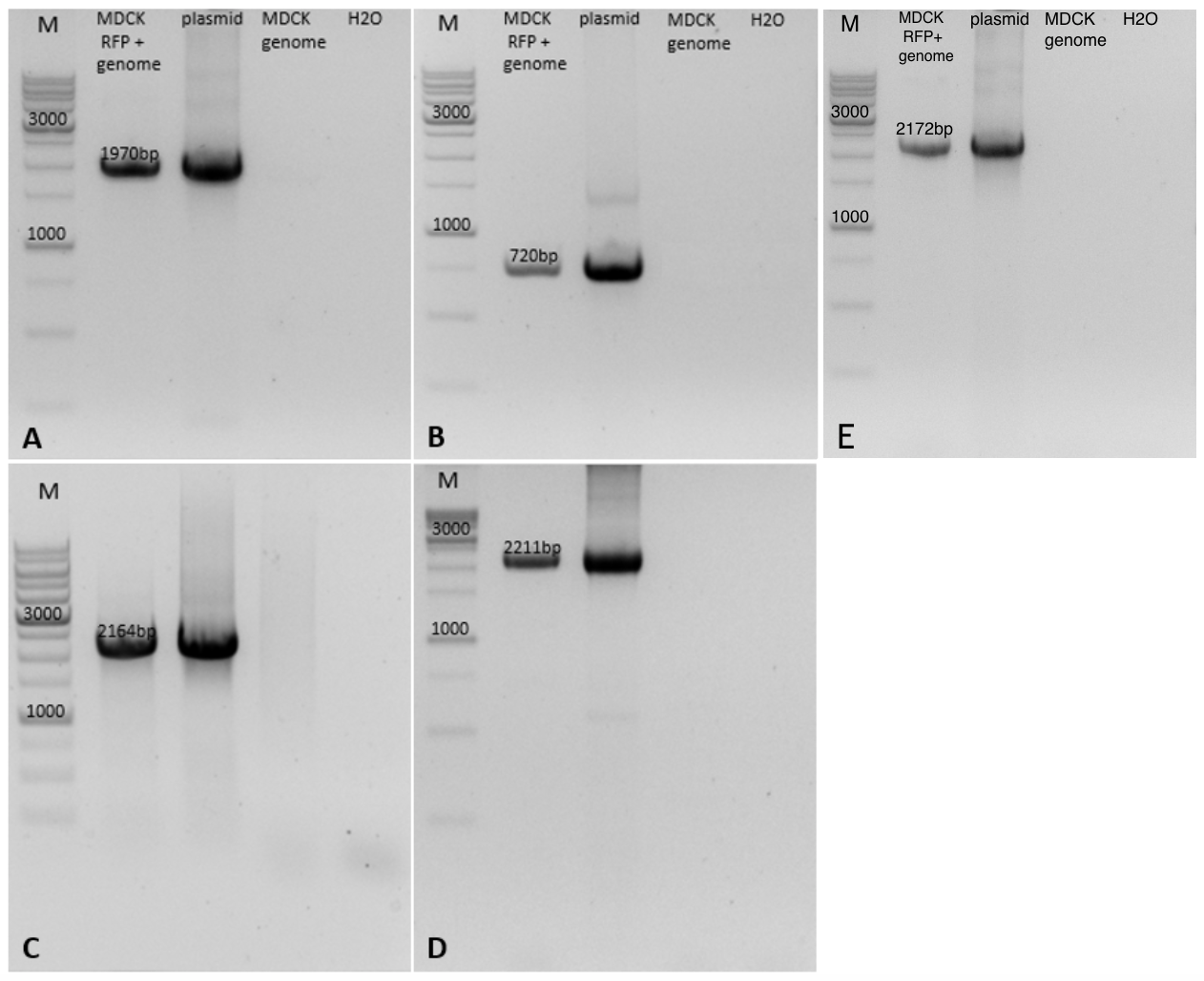

Supplement: Supplementary file 2 [file f1000research-7-16185-s0001.tgz › e5bca7ea-70d0-467a-86c2-e60df5e518e7.png]

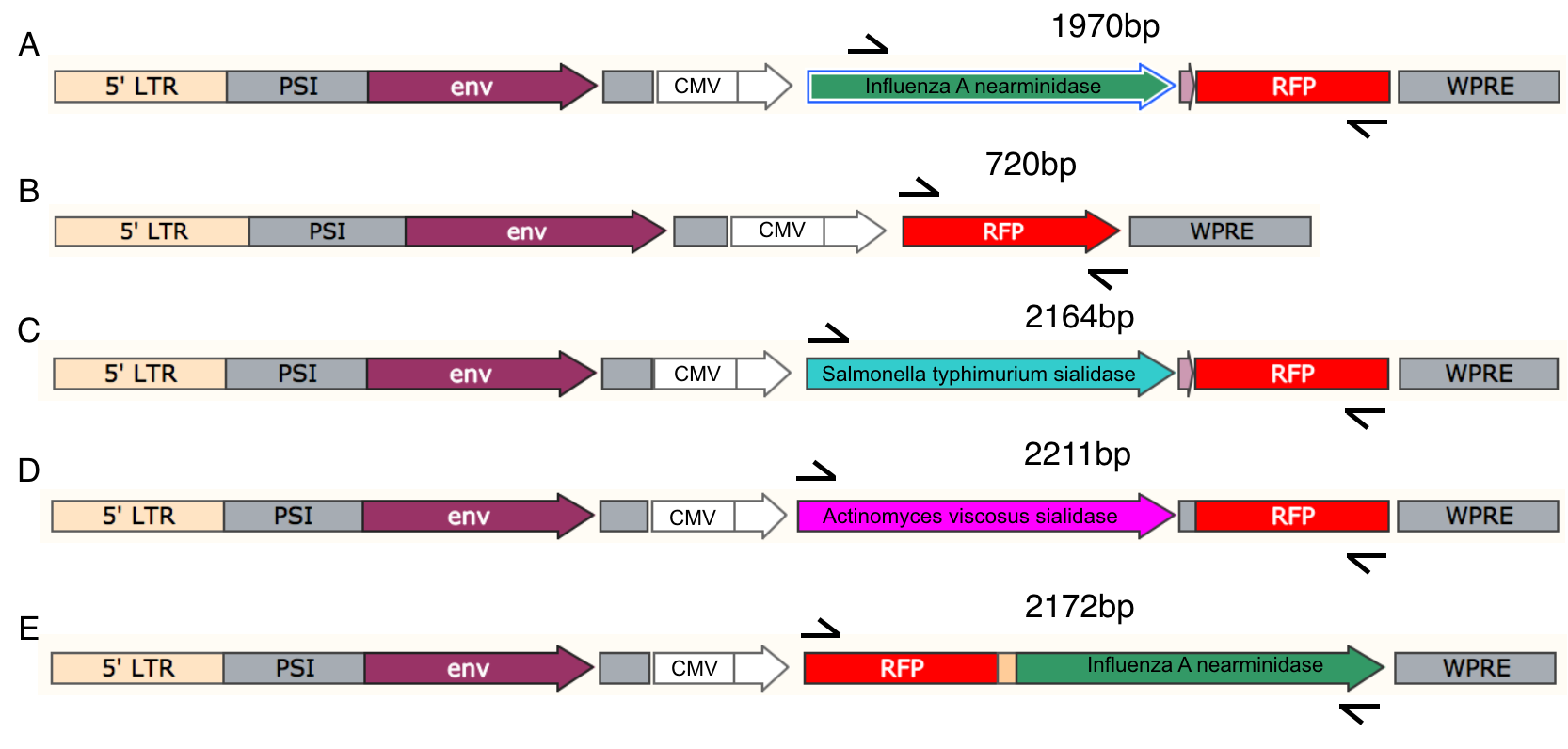

Supplement: Supplementary file 3 [file f1000research-7-16185-s0002.tgz › fd6e8ee8-8309-49d4-aec6-3681c4334f92.png]
