# Supplementary material for: Evaluation of the potential defensive strategy against Influenza A in cell line models
Source: F1000Res. 2018 May 16;7:206. Originally published 2018 Feb 19. [Version 2] doi: 10.12688/f1000research.13496.2 (PMC6008855; doi:10.12688/f1000research.13496.2)
Supplement: Peer review history

# Peer review: Referee response for version 2

**DOI:** 10.5256/f1000research.16185.r34081

**Published:** 2018-06-15

**Authors:** Laurence S. Tiley

**Affiliations:** 1 Department of Veterinary Medicine, University of Cambridge, Cambridge, UK

The authors have responded satisfactory  to the points I raised. They have overdone their response to my suggestion for brief comment about SA-independent entry. I did not mean to suggest that the importance  of SA in influenza entry was in doubt. Overall,the manuscript has been improved to an acceptable standard.

I have read this submission. I believe that I have an appropriate level of expertise to confirm that it is of an acceptable scientific standard.

# Peer review: Referee response for version 2

**DOI:** 10.5256/f1000research.16185.r34082

**Published:** 2018-05-25

**Authors:** Gillian M Air

**Affiliations:** 1 Department of Biochemistry and Molecular Biology, University of Oklahoma Health Sciences Center, Oklahoma City, OK, USA

The authors have made appropriate modifications. The article still contains a few language problems but the meaning is clear.

I have read this submission. I believe that I have an appropriate level of expertise to confirm that it is of an acceptable scientific standard.

# Peer review: Referee response for version 1

**DOI:** 10.5256/f1000research.14654.r32149

**Published:** 2018-04-05

**Authors:** Laurence S. Tiley

**Affiliations:** 1 Department of Veterinary Medicine, University of Cambridge, Cambridge, UK

Summary:

 This paper reports the construction of some vectors to express various sialidases with the ultimate intention of generating transgenic chickens expressing these genes.   The strategy is intended to protect chickens against influenza virus by eliminating the receptor for the virus. The vectors were tested transiently in 293T cells and then in stable MDCK cell lines. Expression of the influenza NA  and S.typhimurium NA reduced alpha 2-3 sialic acid levels at the cell surface as judged by lectin staining.

 I see very little of publishable merit at this stage of the work and the quality of the text requires substantial improvement.

 Specific comments:

 Abstract:

 1.  H5N1  is not showing increasing infection/adaptation for humans.

 2. I suggest the authors consider the increased threat posed by H7N9 which is arguably of far more concern at present.

 Introduction:

 3. The cumulative case count for H5N1 is 454 deaths out of 860 total cases. It is unlikely that the case count of a disease with this high case fatality rate has caused "several thousand" cases and "some with fatal outcome". The wording here is very imprecise.

 4. The citation is from 2005 and totally inappropriate. See: http://www.who.int/influenza/human_animal_interface/2018_03_02_tableH5N1.pdf?ua=1

 5. Seasonal flu vaccination is primarily used to protect individuals in high risk groups. Its impact on  spread in the general population is probably insignificant.

 Methods:

 6. The construction details for  RFP-P2A-infNA are not provided.

 7. The method for the determination of the lentivector  titre is unclear. The analysis by FACS is OK, but what are the authors referring to when they mention "The viral titre corresponded to the number of **colonies** developed at the highest dilution?"

 8. What exactly do they mean by "three serial infections"?

 9. Typo: they used ~1x10 ^4^, not 1x10 ^-4 ^MDCK cells.

 Note: I could not access all  the supplementary info (even on the Harvard Dataverse).

 Results:

 10. d_infNA-RFP is not defined, but presumably it  is the inactive E262D.

 11. Surely a typo for the SD value here: infNA-RFP - 4.725(±983) 

 12. Before making more complex speculations regarding the inactivity of the hNeu3 and Act.v.Sia constructs,  the authors should demonstrate they are expressed in the correct compartment. RFP expression merely shows the mRNA were translated. 

 13. Another typo for the SD: infNA- RFP, +dox - 1.468 (±439)?

 14. The consistent apparent activity of the N1 neuraminidase acting on non-transduced cells, could more simply be an indication that the protein is being secreted. The lack of data on protein localisation does not help with the interpretation. The "slip-stream" explanation hypothesising that many more cells are transduced than were apparent in the photos is possible, but not very credible as this would also need the lentivirus titre/transduction efficiency to be much higher for the infNA vector.

 15.  Little consideration regarding the topology of their constructs has been given.

 Discussion:

 16. The discussion is quite superficial. The authors might like to mention related approaches such as DAS181 and the discussions regarding this general approach ^1^

 17. There are reports that de-sialylation does not completely abrogate influenza infectivity. A point perhaps worth mentioning briefly.

 18. They may wish to mention more about the possible physiological consequences e.g. the potential  impact on the MCE of constitutively expressing a sialidase ^2^

 19. The choice of appropriate promoter is not discussed in any detail. Presumably the intention is to restrict expression to relevant tissues i.e.  the respiratory  and GI tract in the case of poultry. Do they have a particular promoter in mind?  

 20. The practicalities of using  a Dox-inducible promoter are  not considered. Is the intention to treat flocks continuously with Dox to prevent infection,  or to respond prophylactically to an outbreak in a nearby location? The use of Dox in this way is undesirable for several reasons.

I have read this submission. I believe that I have an appropriate level of expertise to confirm that it is of an acceptable scientific standard, however I have significant reservations, as outlined above.

## References

[1] NichollsJMMossRBHaslamSM: The use of sialidase therapy for respiratory viral infections. *Antiviral Res*.2013;98(3) : 10.1016/j.antiviral.2013.04.012 401-9 10.1016/j.antiviral.2013.04.012 23602850PMC7172378

[2] DuezJSixtNPechinotA: Influenza virus infection: don't forget the role of the mucociliary system!. *Journal of Antimicrobial Chemotherapy*.2008;63(2) : 10.1093/jac/dkn468 421-422 10.1093/jac/dkn468 19022776

# Response

**Published:** 2018-05-04

**Authors:** Ekaterina Antonova

**Affiliations:** Moscow Institute of Physics and Technology, Russian Federation

Abstract

 1-2. Agreed. The statement regarding H5N1 was modified to ‘Avian H5N1 and  recently H7N9 virus has a potential pandemic threat.’ in the version 2.

 Introduction

 3.The sentence has been corrected from “Several thousands of cases of human infection were recorded and many of them with a fatal outcome” to “860 cases of disease in human population have been detected since 2003 and 454 of them with fatal outcome (see http://www.who.int/influenza/human_animal_interface/2018_03_02_tableH5N1.pdf?ua=1

 4. The reference has been changed in the version 2.

 5. Agreed. The sentence has been corrected

 Methods

 6. The information has been added in the Methods part: “Additionally the reverse construction RFP-P2A-infNA was cloned in the same vector. (Supplementary Figure 1A)”.

 7. The sentence  "The viral titre corresponded to the number of colonies developed at the highest dilution" was deleted due to it is related to the titre determination with drug selection. However lentiviral constructions with resistant genes were not considered in the publication. Instead, the following text has been added in the version 2: “FACS analysis was performed using non-infected cells as a negative control. The cells with 10%-40% RFP-positive were selected for titer calculation. The titer was calculated using the equation: Titer (Transduction Unit /ml) = (Cell Number in each well used for infection on Day 2 × percentage of GFP/RFP positive cells (should be 10%-40%))/(virus volume used for infection in each well × dilution fold)”

 8. The three repeats were mentioned. The phrase  "three serial infections”  was corrected in the text to avoid confusion: “Infection with the same aliquot was used in three repeats.”

 9. The typo has been corrected in the version 2.

 Please check the supplementary availability now.

 Supplementary Figure 1A & 1B. Genetic constructs. 

Click here to access the data.

 Supplementary Figure 2. Confirmation of inserted genes in MDCK genome after lentiviral transduction.

Click here to access the data.

 Supplementary Figure 3. Schematic of integrated constructs with primers for PCR analysis.

Click here to access the data.

 Results

 10.The mention has been added in the part Methods/Catalytically inactive sialidaseю

 11. Corrected:  4.725(±0.983)

 12. We used  well-known transmembrane domain of Influenza neuraminidase for proper localization of both Act.v.Sia and Sal.t.Sia sialidases on cytoplasmic membrane. The sequence of the transmembrane domain and stem loop was the same for both bacterial sialidases, however only one was active. It is known that the sialidase hNeu3 is localised on the plasma membrane. We cloned the gene and checked the sequence. Lectin binding assay was used to confirm its functionality on the cell surface hence the correct localisation (in the case if activity was demonstrated). However we agree with your point and we have noticed it in the text (Results/Transient expression of genetic constructs in HEK293 cell lines)

 13. Corrected: 1.468 (±0.439)

 14. We have considered the hypothesis regarding secreted neuraminidase and rejected it. First, because we used neuraminidase from Influenza Puerto Rico strain (Addgene). And it is well-known that the neuraminidase is located on the plasma membrane surface. We sequenced the neuraminidase gene after cloning. Second, the reversion of positions of neuraminidase and RFP would not influenced on neuraminidase secretion. But in our case only RFP-positive cells were desialylated (CMV-RFP-P2A-infNA expression) that exclude secretion. Regarding the lentivirus titre/transduction efficiency. The lentivirus titre was defined by RFP fluorescence. However not all RFP sequences could be translated properly due to the effect of termination of translation at the end of 2A peptide that resulted in lower RFP fluorescence. Hence the lentivirus titre could be underestimated. In result the cells were infected by larger quantity of viral particles. FACS data was normalised to RFP fluorescence level when analysing the level of lectin binding. 

 15. We used well-characterised transmembrane domain to target all the enzymes (except human Neu3) on the cell surface and made functional estimation of catalytic activity using lectin binding assay. Enzymatic activity of a protein in the cell expressing reporter confirms membrane location of the protein on the cell membrane.

 Discussion

 16. The mention regarding DAS181 has been added in the  discussion part: “Previously, a recombinant sialidase (DAS181) has already demonstrated its antiviral effectivity in the cell culture (Malakhov MP et al., 2006)) and in vivo, using a mouse model (intranasal injection of the recombinant fusion protein - DAS181) (Belser JA et al., 2007). Thus, the principal possibility of the usage of membrane-anchored  sialidase as antiviral defence has been demonstrated.”

 17.  We added in the discussion points regarding the possible risk: "There are several concerns to use the strategy. The first is that influenza virus receptors may be different from SA, thus, sialidase expression may be ineffective. It has been reported that the virus binding could still occur in the MDCK cell line desialylated with addition in the medium *Micromonospora viridifaciens *sialidase ( Stray *et al.*, 2000). The authors suggested that influenza virus infection can result from sialic acid–independent receptors, either directly or in a multistage process. The presence of sialic acid may enhance virus binding to the cell surface to increase interaction with secondary receptors to mediate entry. Also the authors mentioned that high multiplicity of infection has an increased requirement for sialic acids and desialylation will inhibit the virus amplification. Although the HA of a human H1N1 strain was shown to bind other glycoconjugates (Rapoport *et al.*, 2006), the exact receptors to enter into the cell have not been found. Stray et al., used NA-deficient virus in the research which was obtained in a laboratory. It is unlikely that  such virus would exist in nature. Probably, the barrier to efficient infection and transmission between organisms is due to inefficient use or expression of a yet unidentified entry mediator."

 18. Added: “Another concern of the stable expression of sialidases is disruption of physiological functions required for proper glycosylation ( Gutierrez *et al.*, 1987; Michalek *et al.*, 1988; Pangburn *et al.*, 2000; Varki, 1992). The probable consequences of constitutive expression of membrane sialidase *in vivo*  are not known. It has been shown that overexpression of the human ortholog *NEU3* membrane sialidase under the β-actin promoter in transgenic mice resulted insulin-resistant diabetes mellitus (Sasaki *et al*., 2003). The authors focused on insulin signaling only. However at least the data shows that the constitutive membrane sialidase expression was not lethal for mice. 

 We are going to reduce a probable negative impact on organism using tissue-specific expression of the selected gene. It is necessary to predict a potential impact on the epithelium of intestine and trachea in vivo. There are no similar genetic experiments. However the consequences of tissue-specific expression could be considered from the researches where local desialylation was done. It has been shown that intranasal sialidase treatment does not affect the properties of respiratory mucus, nor did it affect the normal mucus transport activity on ciliated epithelium (King *et al.*, 1974; Meyer *et al.*, 1975). Some authors showed that Influenza infection is associated with an increased risk of secondary infection by *Streptococcus pneumoniae* (Sluijs et al., 2006). There are concerns that, probably, tissue-specific expression of sialidases would result in a greater chance of bacterial infection in the respiratory tract due to exposure of its binding sites that were masked by terminal sialic acid. It has been shown that the binding sites for the bacteria already exist on the normal respiratory surface and sialidase treatment does not introduce de novo bacterial adhesion sites. Also  the risk of infection arises due to damage of mucus and epithelial cells. But it is not clear whether the secondary *S. pneumoniae* infection happens due to viral NA activity or from a secondary effects of influenza virus intracellular amplification. Influenza infection in the respiratory tract causes much deeper changes to the epithelium than treatment of the surface of the epithelial cells with a sialidase, because Influenza infection in humans and in mice results in vast exposure of the ciliated epithelial cells down to the basal cells and the basement membrane (Walsh *et al* , 1961; Plotkowski *et al.*,1986). Thus, the most likely the increased *S. pneumoniae *infection is the result of airway epithelial damage caused by the virus infection but not just desialylation. 

 In the gastrointestinal tract SA is an abundant sugar residue in mucin that is a key target of intestinal bacteria. Expression of sailidases will release free SA from mucins that could drives intestinal inflammation and infection. For example, elevated levels of free sialic acid in the gut, during and post antibiotic treatments, promote the expansion of Clostridium difficile, that utilises free SA. Also it has been shown that sialidase activity can promote the outgrowth of *Escherichia coli* and result in imbalanced microbiota, inflammation, colitis development due to α (2–3)-sialic residues promote expansion of *E. coli *(Huang *et al*., 2015). Nevertheless, we do not know the probable negative impact on the physiology of avian respiratory and digestive tract. Thus, an optimal level of desialylation can be established in the tissue when combining genetic constructs with varying sialidase activity and tissue-specific promoters to avoid  disruption of physiological functions in our approach. Additionally, a gene expression with time limit could prevent a probable negative impact on a living organism. Michael P. Malakhov *et al.* showed that temporary desialylation of respiratory tract during 7 days using recombinant sialidase protein did not cause any symptoms of toxicity or inflammation in ferrets and mice that were not infected by the virus (Malakhov *et al*., 2006). This *in vivo *data support our idea that temporary inducible expression of a sialidase will not have serious negative physiological effects for transgenic animals.”

 19. Yes, we have. We have analysed several promoters specific for intestine and trachea. We have measured expression in scraping of the epithelium from the chicken intestine and trachea by RT-PCR and selected several tissue specific genes that have different expression level.

 Respiratory tract: SFTPA2, SLC34A2. Related expression normalized to GAPDH: SFTPA2 (3,5±0,7); SLC34A2 (0,002). p<0,001

 Digestive tract: RBP2, GUCA2A, MUC13, OLFM4. Related expression normalized to GAPDH: RBP2 (1,1±0,01);

 GUCA2A (0,4±0,005); MUC13 (0,18±0,05); OLFM4 (0,09±0,015), p<0,01.

 20. It is planned to use Dox prophylactically to an outbreak. The practicalities of using Tet-on system were added: “We are planning to use the Tet-on system as a potential model for creation of genetically modified organism that allows to induce a sialidase expression temporary. Transgenic transcription factor rtTA will be expressed under control of a tissue-specific promoter and a sialidase will be expressed under control of TRE promoter. This enables tissue-specific inducible control of the transgene expression and will provide a powerful and flexible resource for studies of influence of sialylation on chicken physiology and on viral infection. The level of sialylation will return to the previous level during a period of time after withdrawal of doxycycline. The required time depends on the sialic acid turnover rate. The inducing agent can be treated prophylactically to genetically modified birds in an outbreak that can protect livestock and locally stop infection spread. It is known that the inducible system has some limitations. Firstly, it has basal transgene ‘leak’. However in our case at least the leak will be in the specific tissue.  The probable consequences of the leak will be studied. Secondly, the feeding of domestic birds with the doxycycline antibiotic can compromise its safety as biological products. However it is a temporary measure that could help to save the stock. The efficacy of our strategy needs to be evaluated *in vivo* to resolve all the concerns.'

# Peer review: Referee response for version 1

**DOI:** 10.5256/f1000research.14654.r31110

**Published:** 2018-03-12

**Authors:** Gillian M Air

**Affiliations:** 1 Department of Biochemistry and Molecular Biology, University of Oklahoma Health Sciences Center, Oklahoma City, OK, USA

The authors have expressed membrane-anchored constructs of influenza, human and bacterial sialidases in cells and measured the effect on cell surface sialylated glycans. The study is preliminary but well executed as far as it goes.

 General comments:

1. The manuscript does not include evaluation of influenza virus infection so the title is exaggerated. Maybe “Evaluation of *potential* defense strategy…”. would be less misleading.
2. The Figure legends are very terse and a reader needs to make some guesses at what is being shown. For example, what are the boxes on the panels in Figures 3 and 4?
3. The manuscript needs to be checked for spelling. “sialilation” appears several times and there are other errors such as S. typhimyrium on p.7.

 

 Specific comments:

1. Abstract: I have not seen data that shows the H5N1 virus is increasingly infective for humans. Either reference this statement or modify it.
2. Abstract and text: the increased activity seen with influenza neuraminidase is likely due to the construct being more native and better folded. The S. typhimurium sialidase itself has much higher activity than influenza NA.
3. P.5: the Maakia amurensis lectins have been variable between suppliers and one form binds additional  glycans, as has been discussed in several recent publications. More information is needed to be sure the lectin used has alpha2-3 specificity.
4. P.5, first paragraph of Results. “The optimal sialic acids level on the cells surface”. I think this should refer to sialidase, not sialic acid.
5. P.5 The influenza NA does indeed cleave alpha2-6 linked sialic acid but at 1/3 to 1/5 the rate of alpha2-3.
6. P.5: while the assay to show reduction of cell surface sialic acids fits the goal of the project, it might be useful to carry out classical enzyme assays using the expressed sialidases to determine if the constructs used have similar activity to their wild type counterparts and if not, if they can be improved.

I have read this submission. I believe that I have an appropriate level of expertise to confirm that it is of an acceptable scientific standard, however I have significant reservations, as outlined above.

# Response

**Published:** 2018-05-03

**Authors:** Ekaterina Antonova

**Affiliations:** Moscow Institute of Physics and Technology, Russian Federation

Respond to the general comments:

 1.The correction has been done in the version 2.

 2.Figure legends have been supplemented with details in the version 2.

 3. The manuscript has been checked for spelling and found errors and typos were corrected in the version 2.

 Respond to the specific comments:

 1.The statement was modified to ‘Avian H5N1 and  H7N9 virus has a potential pandemic threat’ in the version 2.

 2.The information was added in the Results/Transient expression of genetic constructs in HEK293 cell lines.

 3.Maakia amurensis lectins from bioWORLD that have declared alpha(2;3) specificity were used as analogous to an avian hemagglutinin due to they share similar structural fold. 

 The lectins were used for preliminary study and it is planned to continue the research with an avian virus.

 4. Both statements make sense, however “the optimal sialic acids level” is the result of sialidases expression and it undermines not only the level of sialidases on the cell surface but their catalytic activity.

 5.The statement «Unlike human HA that preferentially recognises α (2–6), the viral NA of H1N1 has cleavage activity to both (2-6) and α(2–3) types of linkages» was supplemented with the information that NA also cleaves alpha2-6 linked sialic acids at lower rate than alpha2-3 .

 6.The aim of the project was to compare created genetic constructs between each other and use some of them in future researches.  We didn’t try to achieve similar activity of the constructs to their recombinant counterparts. However it would be interesting to check how folding and localisation influence on the enzymes activity.
